# Effects of Mind–Body Exercises (Tai Chi/Yoga) on Heart Rate Variability Parameters and Perceived Stress: A Systematic Review with Meta-Analysis of Randomized Controlled Trials

**DOI:** 10.3390/jcm7110404

**Published:** 2018-10-31

**Authors:** Liye Zou, Jeffer Eidi Sasaki, Gao-Xia Wei, Tao Huang, Albert S. Yeung, Octávio Barbosa Neto, Kevin W. Chen, Stanley Sai-chuen Hui

**Affiliations:** 1 Department of Sports Science and Physical Education, The Chinese University of Hong Kong, Shatin, Hong Kong, China; liyezou123@cuhk.edu.hk; 2 Department of Sport Sciences, Institute of Health Sciences, Federal University of Triangulo Mineiro, Uberaba, MG 38025-440, Brazil; jeffersasaki@gmail.com (J.E.S.); octavio.neto@uftm.edu.br (O.B.N.); 3 Key Laboratory of Behavioral Science, Institute of Psychology, Chinese Academy of Sciences, Beijing 100101, China; weigx@psych.ac.cn; 4 Department of Physical Education, Shanghai Jiaotong University, Shanghai 200240, China; taohuang@sjtu.edu.cn; 5 Depression Clinical and Research Program, Harvard Medical School, Boston, MA 02114, USA; ayeung@mgh.harvard.edu; 6 Center for Integrative Medicine, School of Medicine, University of Maryland, Baltimore, MD 21201, USA; kchen@umaryland.edu

**Keywords:** Yoga, Tai Chi, mindfulness, psycho-social stress, HRV, autonomous nervous system

## Abstract

Background: Heart rate variability (HRV) as an accurate, noninvasive measure of the Autonomous Nervous System (ANS) can reflect mental health (e.g., stress, depression, or anxiety). Tai Chi and Yoga (Tai Chi/Yoga), as the most widely practiced mind–body exercises, have shown positive outcomes of mental health. To date, no systematic review regarding the long-lasting effects of Tai Chi/Yoga on HRV parameters and perceived stress has been conducted. Objective: To critically evaluate the existing literature on this topic. Methods: Five electronic databases (Web of Science, PubMed, Scopus, SportDiscus and Cochrane Library) were searched from the start of the research project to July 2018. Study selection, data extraction, and study quality assessment were independently carried out by two reviewers. The potentially identified randomized controlled trials (RCT) reported the useful quantitative data that were included only for meta-analysis. Results: meta-analysis of 17 medium-to-high quality RCTs showed significantly beneficial effects on HRV parameters (normalized low-frequency, Hedge’s g = −0.39, 95% CI −0.39 to −0.56, *p* < 0.001, I2 = 11.62%; normalized high-frequency, Hedge’s g = 0.37, 95% CI 0.22 to −0.52, *p* < 0.001, I2 = 0%; low-frequency to high-frequency ratio, Hedge’s g = −0.58, 95% CI −0.81 to −0.35, *p* < 0.001, I2 = 53.78%) and stress level (Hedge’s g = −0.80, 95% CI −1.17 to −0.44, *p* < 0.001, I2 = 68.54%). Conclusions: Stress reduction may be attributed to sympathetic-vagal balance modulated by mind–body exercises. Tai Chi/Yoga could be an alternative method for stress reduction for people who live under high stress or negative emotions.

## 1. Introduction

Heart rate variability (HRV) is a noninvasive index to measure the psycho-physiological phenomenon of oscillation in the time intervals between consecutive heartbeats [1]. It has been increasingly applied in both clinical settings and research fields for its ability to monitor the dynamic equilibrium between sympathetic and parasympathetic nervous activity [1]. Many clinicians and researchers believe that HRV is not only a predictor of mortality in patients with post-myocardial infarction and heart failure [2], but that it can also be used to objectively assess emotional health especially stress levels [3,4]. Generally, a high resting HRV reflects good health and high tolerance for stress or resilience [5], while reduced HRV is associated with higher risk to develop mental illness as well as a slow recovery process [6]. Long-term participation in exercise training has been shown to induce a resting bradycardia accompanied by reduced sympathetic activity and/or elevated parasympathetic activity and a marked reduction in intrinsic heart rate [7]. Additionally, increasing evidence indicates that regular exercise could reduce an individual’s stress level and increase well-being as well [8]. Based on this principle, regular physical exercise may potentially generate an optimized HRV for psychosomatic well-being and alleviate stress state.

Tai Chi and Yoga (Tai Chi/Yoga) are two of the most popular mind–body exercises, practiced by all age groups with different health conditions around the world, for health promotion and symptomatic management [9,10]. Tai Chi and Yoga originated in China and India, respectively [11,12]. Compared to conventional exercises that usually focus on muscular strength and endurance, Tai Chi/Yoga share similar elements: training involves mind–body cultivation through slow voluntary movements, full-body stretching and relaxation, diaphragmatic breathing practice, meditative state of mind and mental concentration [13,14,15,16,17]. Given that Tai Chi/Yoga are easily accessible and easy-to-learn, researchers have recently paid considerable attention to investigate their effects on health outcomes. Although it has been well-established that Tai Chi/Yoga training could induce improvements of self-reported outcomes of stress and objective physical function [18,19,20,21], it still remains unclear whether Tai Chi/Yoga could effectively enhance HRV. HRV is not usually viewed as an intended outcome. Rather, it is of great significance to consider HRV as an interesting moderator between Tai Chi/Yoga and emotional outcomes.

In several early observational studies [22,23,24,25,26] with cross-sectional design, the results showed that Tai Chi/Yoga has the potential to enhance HRV through increased parasympathetic modulation [22,23] and/or to reduce sympathetic activity [24,25,26]. To further substantiate the potential beneficial effect of prolonged Tai Chi/Yoga training for autonomic nervous function, a growing number of experimental studies have recently been conducted [27,28,29,30,31,32,33]. As the number of trials increases, two research groups attempted to systematically evaluate the existing literature regarding the effects of Tai Chi [34] or Yoga [35] on HRV. However, they either used qualitative synthesis [34] or meta-analyzed data about expiratory-to-inspiratory ratio, with inclusion of all age group and acute effects of yoga [35]. In particular, study findings of the meta-analysis [35] were not significantly supportive of the effects of Yoga on the selected HRV parameter. It is largely unknown whether effects of chronic Tai Chi/Yoga training on the commonly used time–domain or frequency–domain measurements of HRV. Moreover, there is no systematic study to evaluate the effect size of chronic Tai Chi/Yoga on stress. We therefore conducted a systematic review to quantitatively synthesize the existing literature on this topic. Study findings of this review may provide clinicians and researchers with some suggestions about both applications of HRV for both clinical settings and exercise interventions.

## 2. Methods

### 2.1. Literature Search

The Preferred Reporting Items for Systematic Reviews and Meta-Analysis guideline was employed to report the current meta-analysis [36]. Five English-language databases (Web of Science, PubMed, Scopus, SportDiscus and Cochrane Library) were systematically searched from the start of the research project to July 2018. Mind–body exercise (Tai Chi, Tai ji, or Yoga) was combined with heart rate variability, HRV, autonomic nervous system, and cardiac vagal tone. To obtain as many relevant studies as possible, reference lists of initially identified documents meeting the inclusion criteria were manually searched. Both electronic and manual literature search were performed by the first author of this review (L.Z.).

### 2.2. Inclusion Criteria and Study Selection

Only randomized controlled trials published in English-language peer-review journals were considered in this review. Studies were included if they met the following inclusion criteria: (1) Study participants had to be 18 years old or above when they partook in mind–body exercise intervention; (2) Tai Chi or Yoga were the main intervention modalities and could not be performed in a heated environment; (3) Intervention duration of Tai Chi or Yoga had to be at least three weeks in duration to determine the long-lasting effects on autonomic nervous function; (4) Participants of control groups either kept their unaltered lifestyle or received other active control conditions; (5) At least one resting HRV (5 min or above) was recorded regardless of measuring position (sitting, standing, or lying); and (6) Power spectral analysis involved either auto-regressive modeling or Fourier type transformation. We did not limit the HRV metrics such as time–domain (e.g., SDNN, RMSSD, RR interval, pNN50) and frequency–domain (e.g., low-frequency, LF; high-frequency, HF; LF/HF ratio; normalized low-frequency, nLF; normalized high-frequency, nHF). As mentioned previously, Yoga failed to show favorable effects compared to control conditions on expiratory-to-inspiratory ratio in the latest meta-analysis [35], so studies reporting this index were excluded. In those eligible trials where abnormal or ectopic beats were clearly described, they were only considered for data analysis. Initially, two reviewers (L.Z. and K.C.) independently performed study selection against the inclusion criteria. If any disagreement regarding the eligibility of identified studies occurred between the two reviewers, a third reviewer (A.Y.) was invited to discuss with them until consensus was reached.

### 2.3. Methodological Quality Assessment of Randomized Controlled Trials

The Physiotherapy Evidence Database (PEDro) Scale was employed to complete appraisal of methodological quality of all eligible trials. It consists of 11 items as follows: eligibility criteria, random allocation, allocation concealment, baseline equivalence, participant blinding, instructor blinding, assessor blinding, retention rate ≥ 85%, intention-to-treat analysis for missing data, between-group statistical comparison, and point measure/measure of variability ≥ one key outcome.

Item 1 (eligibility criteria) is related to the external validity or generalizability of trials selected, whereas Item 2 to 9 involves internal validity appraisal of all eligible trials. In addition, Item 10 and 11 involves result interpretation. Considering the reality that blinding of participants and instructor (s) were unattainable among exercise intervention studies, these two items were removed, leading to a final number of 9 items. We examined each individual item to objectively evaluate risk of bias (e.g., selection, performance, detection, or attrition) across trials, but did not summarize methodological quality of each trial according to its sum score. The study quality appraisal is similar to study selection administered by two independent reviewers (L.Z. and K.C.) and a third reviewer (A.Y.) was used when necessary.

### 2.4. Data Extraction and Analysis

Two reviewers independently extracted data from each eligible trial where it included study characteristics (study reference), characteristics of participants, intervention protocol, outcome measured, and safety (adverse event) during mind–body exercise intervention. Information about the characteristics of participants (sample size, gender, mean age, health status, and attrition rate) were extracted, which makes the results of this review interpretable or generalizable. Information about the intervention protocol were extracted, including intervention duration, weekly training frequency, and session length. Time–domain and frequency–domain measures were primary outcomes and psychological-related parameters were also included. Given that the length of recording period, detection methods, and recording positions (sitting, lying, or supine) could affect evaluation of heart rate variability, these factors were also included in Table 1. In addition, we had contacted the authors of an RCT by Lin et al. [37] to request data (LH, HF, LF/HF ratio), but as of yet, we have not received feedback. Given that the parameter of “perceived stress” was reported in this study, we included it for meta-analysis on this behavioral outcome, but not HRV parameters.

We meta-analyzed data using the Comprehensive Meta-Analysis Version 3. As suggested by Borenstein et al. [36], only if at least four studies reported the same HRV or psychological outcome (stress, depression, or anxiety), its pooled effect size (based on Mean and SD at both baseline and post-intervention of each group) was calculated with a conservative random-effects model. We categorized the magnitude of intervention effect, with Hedge’s g of 0.2 to 0.49 indicating small effect, 0.5 to 0.79 indicating moderate effect, and 0.8 and above indicating large effect). Statistical data (mean and standard deviation at baseline and post-intervention along with the number of participants of each group at post-intervention) were input for meta-analysis. If data were missing in a manuscript, we contacted the corresponding author via email for data request. The Q statistic (small = 25%, moderate = 50%, and 75% = large) was used to determine whether heterogeneity existed across studies. In any study where researchers attempted to compare mind–body exercise intervention with two control conditions, to reduce the unit-analysis error, the sample size of experimental group was equally distributed for two comparisons while its mean and standard deviation remained unaltered. We used the Egger’s regression intercept test to detect publication bias, along with the visual funnel plot.

## 3. Results

### 3.1. Eligible Study Selection

**Figure 1:**
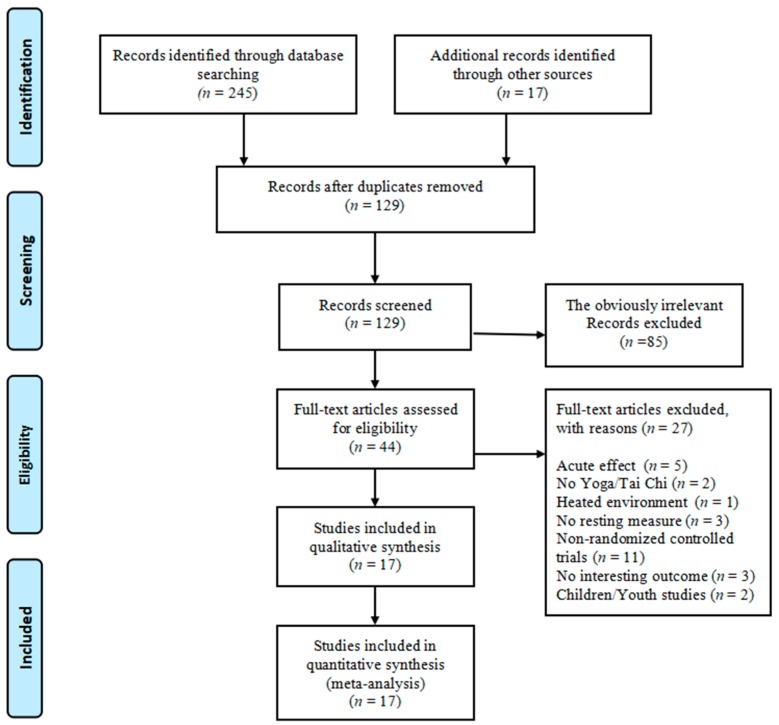
The process of selecting randomized controlled trials.

Two-hundred and sixty-two records were retrieved from five electronic databases and other sources. After duplicates were removed, 129 documents were further assessed based on the titles and abstracts of articles, leading to 85 irrelevant articles excluded. Based on the pre-determined inclusion criteria, we performed full-text article assessment. Of 44 full-text articles, 27 were excluded because researchers investigated the acute effects of mind–body exercise (*n* = 5), no Tai Chi/Yoga was implemented as the primary intervention (*n* = 2), Yoga training took place at a heated environment (*n* = 1), non-randomized controlled trials were used (*n* = 11), outcome of interest was not reported (*n* = 3), children or youth were included as study participants (*n* = 2), and resting measure of HRV was not performed (*n* = 3). Therefore, we included 17 RCTs in this review [38,39,40,41,42,43,44,45,46,47,48,49,50,51,52,53]. The process of study selection is presented in Figure 1.

### 3.2. Study Characteristics

Study characteristics of 17 eligible RCTs (Tai Chi = 4 and Yoga = 13) are summarized in Table 1. The earliest study regarding the effects of mind–body exercise (Yoga) on HRV was published in 1997 [49], followed by a study in 2009 [48]. The remaining studies were published between 2012 and 2018. While 12 RCT involved healthy individuals, five other studies reported people with disease (low back pain, chronic heart failure, nasopharyngeal carcinoma, or fibromyalgia) [41,50,51,52,53]. Sample size (attrition rate from zero to 46.8%) varied greatly across studies, ranging from 26 to 355 (mean age ranged from 25.85 to 71.4). Mind–body exercise intervention duration lasted 6 to 19 weeks, the number of weekly sessions ranged from one to seven (each session length took 30 to 90 min). Control group involved either passive (unaltered lifestyle, waitlist, watching TV) or active condition (usual care/standard care, bicycle training, standard prenatal exercise, mindfulness training, usual physical activity, stretching, or other gym exercise). Given the safety of patients in two studies, researchers allowed all patients to receive chemoradiotherapy [50] or standard medical therapy [52]. Electrocardiogram was the most commonly used tool for HRV measure, with recording length of ≥5 min. Of 17 studies, no adverse events occurred during mind–body exercise intervention.

### 3.3. Methodological Quality of Randomized Controlled Trials

Methodological quality of 17 RCTs were assessed using the adapted PEDro scale. Sum scores across studies ranged from five to nine points, indicating fair-to-high quality. Taking into account each individual item, allocation concealment was only used in six studies [37,38,43,48,50,53] while high retention rate of ≥85% was found in nine studies [39,40,41,42,44,46,48,49,52]. Other points were deducted due to the lack of blinded assessors [38,39,42,44,47,49,50,52] and intention-to-treat analysis [39,40,41,42,44,46,48,49,52]. Baseline equivalence [37] and between-group comparison [40] were each absent in one study, respectively (Table 2).

### 3.4. Effects of Mind–Body Exercises on Psycho-Physiological Parameters

As mentioned previously, only outcomes that were reported in at least four studies were meta-analyzed. In addition, because reporting of HRV components (LF, HF, LF/HF ratio, nLF, nHF, nLF/nHF ratio) varied greatly across studies, the most commonly used measures were selected for meta-analysis. Based on this principle, the nLF (sympathetic and vagal modulation), the nHF (vagal modulation), and LF/HF ratio (sympathovagal balance) were finally considered for meta-analysis.

**Figure 2:**
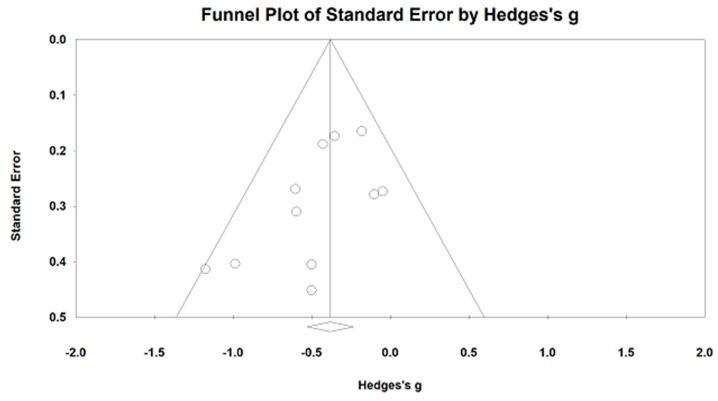
Funnel plot of publication bias for low-frequency power.

**Figure 3:**
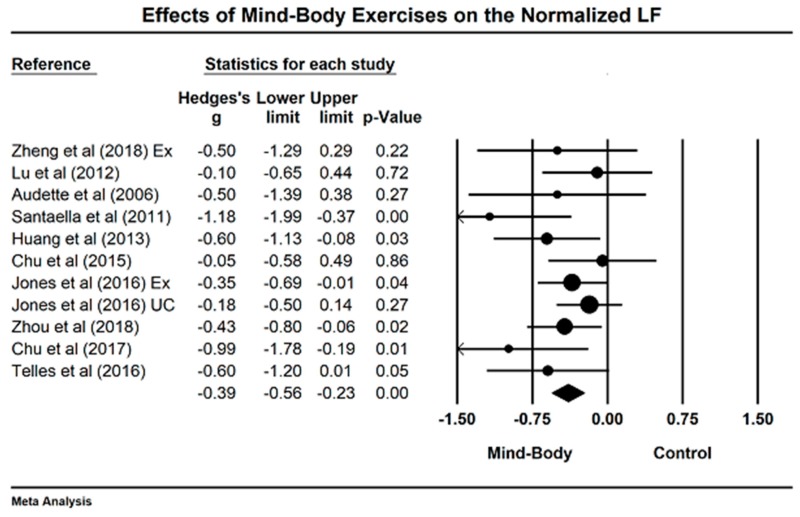
Effects of mind–body exercise on the normalized low-frequency power (LF = low frequency power; Ex = Exercise control group, UC = usual care).

We meta-analyzed the data (12 studies including 14 comparisons) regarding the effects of mind–body exercises on the nLF (greater negative value indicating better sympathetic and vagal modulation). The funnel plot is visually asymmetrical because three outlying comparisons (values of Hedge’s are close to −2) [38,41,52], with Egger’s regression intercept = −3.04, *p* = 0.028. After removing three outliers, the funnel plot (Egger’s regression intercept = −1.73, *p* = 0.07) is symmetrically observed in Figure 2. The study results of remaining studies indicate that mind–body exercises significantly decreased the nLF, as compared to other control groups (Hedge’s g =−0.39, 95% CI −0.39 to −0.56, *p* < 0.001, I^2^ = 11.62%, Q test = 11.62; Figure 3).

**Figure 4:**
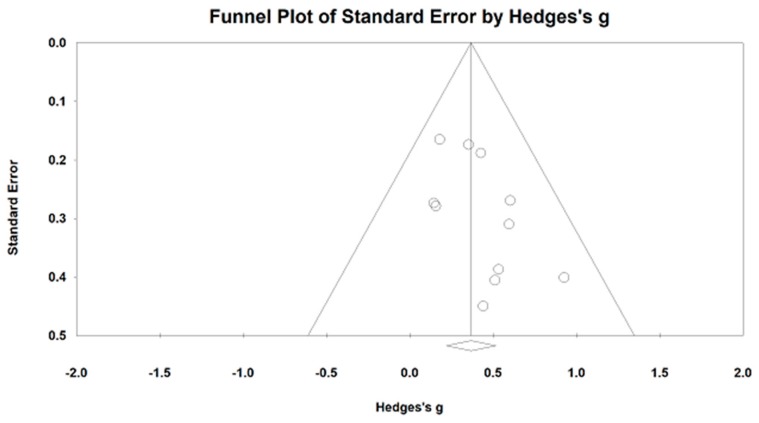
Effects of mind–body exercise on the normalized high-frequency power.

**Figure 5:**
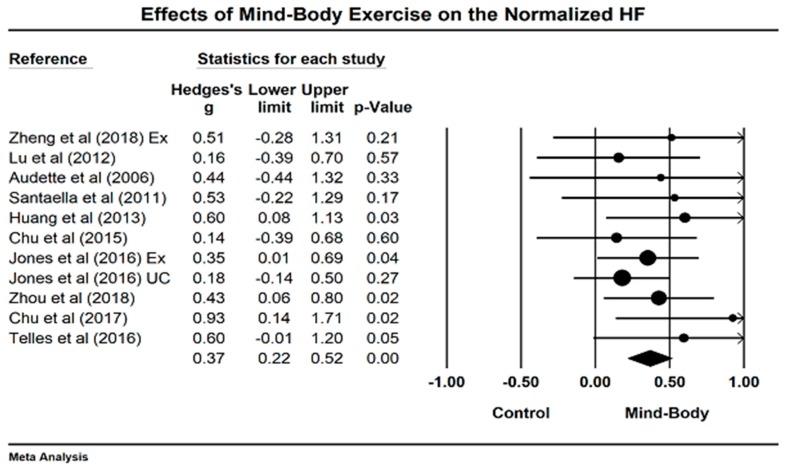
Effects of mind–body exercise on the normalized high-frequency power (Ex = exercise control, UC = usual care).

For nHF, we meta-analyzed the data from 12 studies (14 comparisons). An asymmetric funnel plot was visually observed and presented two outlying studies [38,41,52]. After removing the outliers, the funnel plot is symmetrically presented in Figure 4. The study results of remaining studies indicate that mind–body exercises significantly increased the nHF, as compared to other control groups (Hedge’s g = 0.37, 95% CI 0.22 to −0.52, *p* < 0.001, I^2^ = 0%, Q test = 6.22; Figure 5).

**Figure 6:**
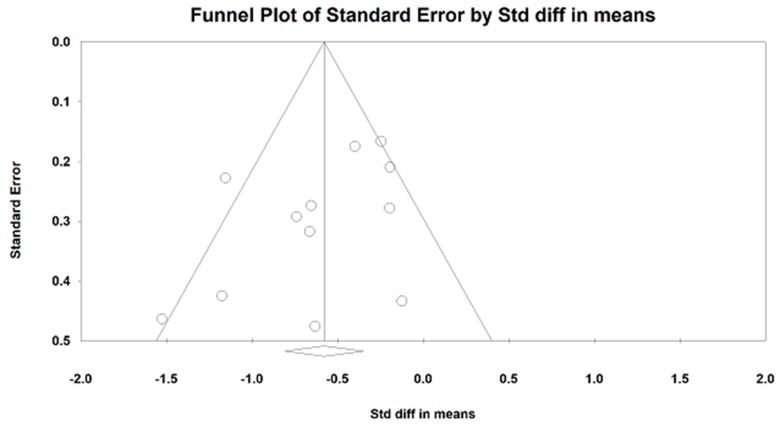
Funnel plot of publication bias for low-frequent to high-frequency ratio.

**Figure 7:**
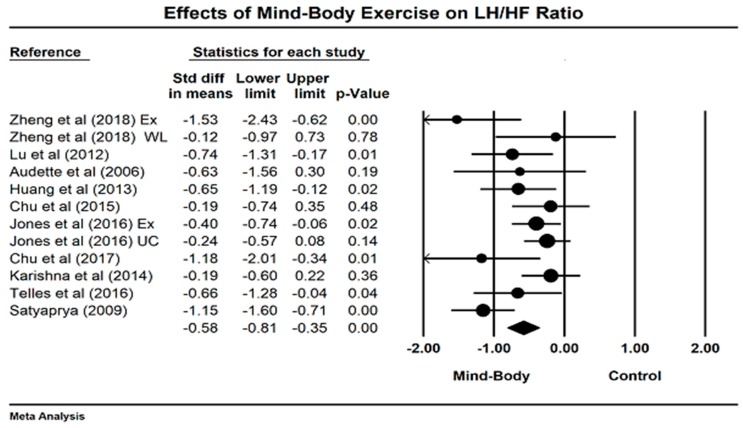
Effects of mind–body exercises on low-frequency to high-frequency ratio (LF/HF ratio = low frequency to high frequency ratio: Ex = Exercise control, WL = waitlist, UC = usual care).

For LF/HF ratio, we meta-analyzed the data from 11 studies (because two studies include two control groups, the number of comparisons is 13 in total), with greater negative value indicating better sympathovagal balance. We visually detected an outlying study (Hedge’s g = −5) based on the Funnel plot along with the Egger’s Regression Test (Egger’s regression intercept = −3.74, *p* = 0.018). After removing this outlier, the symmetric funnel plot (Egger’s regression intercept = −1.96, *p* =0.16) is presented in Figure 6. The results of the meta-analysis indicate that a significant benefit in favor of mind–body exercises on modulating sympathetic-vagal balance in adults (Hedge’s g = −0.58, 95% CI −0.81 to −0.35, *p* < 0.001, I^2^ = 53.78%, Q test = 23.8; Figure 7). Because more than ten studies investigated the effects of mind–body exercises on the LF/HF ratio, we performed sub-analyses for health status and style of mind–body exercise. No significant difference was observed between healthy individuals and people with diseases (chronic low back pain, chronic heart failure, and depressive symptoms) as well as between Tai Chi and Yoga, so we do not present the study results in the text. A meta-regression was performed for weekly total time. It was observed that a significant effect for weekly training time in Tai Chi/Yoga (*β* = −0.00086, 95% CI −0.00234 to −0.00063, *p* = 0.013); A negative relationship was observed between weekly training time and LF/HF ratio.

**Figure 8:**
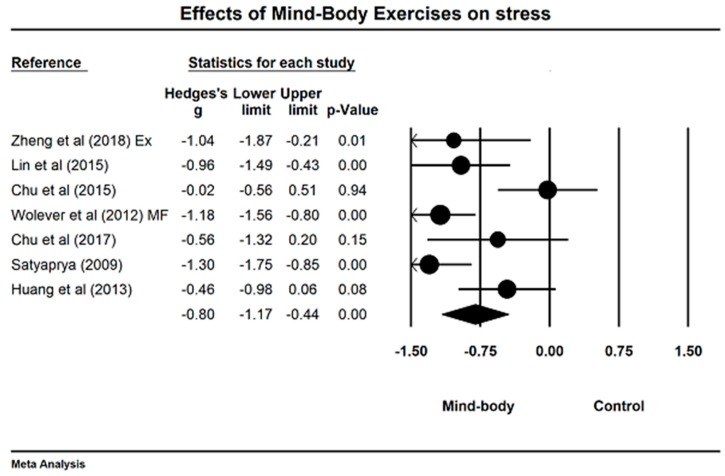
Effects of mind–body exercises on stress reduction (Ex = exercise control, MF = mindfulness control).

We meta-analyzed the data for stress (7 studies with 9 comparisons). Following the sensitivity analysis for stress-related studies, we removed two outlying comparisons [38,47], the study results of meta-analysis indicate that mind–body exercises significantly reduced stress level (Hedge’s g = −0.80, 95% CI −1.17 to −0.44, *p* < 0.001, I2 = 68.54%; Figure 8).

## 4. Discussion

The aim of this meta-analysis was to examine the effects of Tai Chi/Yoga training on the parameters of heart rate variability and perceived stress. The results indicated a small effect of Tai Chi/Yoga training on nLF and nHF, a moderate effect on LF/HF ratio, and a large effect on perceived stress. The literature included in this meta-analysis suggests that the evidence for beneficial effects of Yoga on HRV is more robust than that for Tai-Chi. Overall, it appears that as few as two sessions of 30min/week of Yoga may increase nHF, and reduce both nLF and LF/HF ratio.

### 4.1. Effects of Mind–Body Exercises on Normalized Low-Frequency Power

Overall, the intervention effect was statistically significant but of small magnitude, as denoted by the Hedge’s value. Of the 11 studies included in the final meta-analysis, five demonstrated significant reductions of nLF [42,44,46,50,51]. Participants for these studies were older adults [42], middle-aged women [44], menopausal women [46], patients with nasopharyngeal cancer [50], and sedentary young women [51]. Four of the studies reporting significant results used Yoga as the intervention activity [42,44,46,51], with the total weekly training volume ranging from 60 to 200 min/week, training frequency of 1 to 2 times/week, and total intervention duration of 8 to 16 weeks. The only study demonstrating significant effects of Tai-Chi on nLF was that by Zhou et al. [50], with the training protocol consisting of five 60-min sessions/week for 19 weeks.

Both Tai-Chi and Yoga involve control of body tension and mental concentration, and this presumably promotes reductions in stress [13,14,15,16,17]. These activities may reduce nLF by lowering sympathetic drive and increasing parasympathetic drive of the heart. The number of studies supporting the positive effects of Yoga on nLF were superior than those for Tai-Chi. Among the studies examining Yoga, the results suggest that as little as 30 min of Yoga twice a week may be effective in promoting reductions in nLF [42]. For Tai-Chi, there is a clear need for further studies to examine whether Tai-Chi can promote significant reductions in nLF or not.

### 4.2. Effects of Mind–Body Exercises on Normalized High-Frequency Power

The meta-analysis indicated an overall significant increase in nHF as a result of Tai Chi/Yoga training. This increase was of small magnitude, as characterized by the Hedge’s value. Of the 11 studies meta-analyzed, four demonstrated significant increases in nHF resulting from Tai Chi/Yoga training [44,46,50,51]. These studies included middle-aged women [44], menopausal women [46], patients with nasopharyngeal cancer [50], and sedentary young women [51] as participants. Of the four studies, three utilized Yoga [44,46,51] as the intervention activity while only one included Tai-Chi [50] as the intervention modality. For the studies including Yoga as the intervention activity [44,46,51], total weekly training volume, training frequency, and total intervention duration ranged from 60 to 200 min/week, 1 to 2 times/week, and 8 to 16 weeks, respectively. The study including Tai-Chi [50] as the intervention activity utilized five 60-min sessions/week for a total intervention duration of 19 weeks.

Yoga and Tai-chi are exercise modalities that have a high demand on breathing control [8]. It is well-established in the literature that respiratory rhythm is strongly synchronized with the nHF component of HRV [54]. Tai Chi/Yoga training may promote chronic adaptations in breathing pattern and changes in parasympathetic activity that may be responsible for increases in nHF. While both modalities may potentially increase the nHF component of HRV, the results of this systematic review are controversial, as only four of the 11 studies (3 with Yoga and 1 with Tai-Chi) reported significant results. Thus, further studies on the topic are necessary for drawing more robust conclusions.

### 4.3. Effects of Mind–Body Exercises on LF/HF Ratio

The overall effect of Tai Chi/Yoga training on LF/HF ratio was moderate. Seven of the 12 studies meta-analyzed demonstrated significant reductions in the LF/HF ratio [38,39,44,46,48,51,53]. Participants for these studies were healthy young adults [38], middle-aged and older adults [39], middle-aged women [44], menopausal women [46], pregnant women [48], young sedentary women with depressive symptoms [51], and patients with chronic low back pain [53]. Five of these studies implemented Yoga as the intervention modality [44,46,48,51,53]. In these studies, total weekly training volume ranged from 90 to 360 min/week, training frequency ranged from 1 to 3 times/week, and total intervention duration ranged from 8 to 16 weeks. The two studies that found significant results for Tai-Chi [38,39] employed weekly training volumes of 280 and 300 min/week, training frequencies of 5 and 7 times/week, and total intervention duration of 12 weeks.

Similar to the other HRV outcomes, the results for LF/HF ratio indicated stronger evidence of beneficial effects for Yoga (5 studies) compared to Tai-Chi (2 studies). These results are in line with the reductions observed for nLF and increases observed for nHF, reported earlier. The reductions in LF/HF ratio might be due to a shift to parasympathetic dominance as a consequence of Tai Chi/Yoga training. This is usually the case when changes in nLF are due to reductions in sympathetic drive, and the changes in nHF are due to increases in parasympathetic activity [55,56]. Yet, there is always the possibility that other factors may have been responsible for the changes in nLF and nHF, and, consequently, in LF/HF ratio. Of note, as weekly training time increases, larger effect sizes were observed. Such results indicated that regular training in Tai Chi/Yoga can generate positive effect on LF/HF ratio.

### 4.4. Effects of Mind–Body Exercises on Perceived Stress

The meta-analysis indicated an overall large effect of Tai Chi/Yoga training on perceived stress. Four of the seven studies included in the meta-analysis demonstrated significant reductions in perceived stress [37,38,47,48]. For these studies, participants were healthy young adults, mental health professionals, highly stressed employees, and pregnant women. Of the studies reporting significant results, three employed Yoga [37,47,48] and one employed Tai-Chi [38] as the intervention modality. In the studies with Yoga [37,47,48], weekly training volume ranged from 60 to 360 min/week, whereas training frequency and total intervention duration ranged from 1 to 5 times/week and from 12 to 16 weeks, respectively. For the study using Tai-Chi [38], the training protocol consisted of five 60-min sessions/week during a 12 week period.

It is known that Tai-Chi and Yoga are exercise modalities with slow movements and static stances, which poses a great demand on mental concentration and breathing control [8]. For those skilled Tai Chi/Yoga practitioners, they frequently achieve a meditative state of mind by adjust breathing model and bodily balance [8,13]. All of these features are favorable factors to help concentrate on the moment and momentarily forgetting stressful events of the daily life. In addition, Tai Chi/Yoga are typical aerobic exercises, which was confirmed to have beneficial outcomes for reducing negative affect (REFs). As in any other modalities, there are many physiological processes linked to physical and emotional pleasure that may take place while performing Tai-Chi and Yoga, including hormonal release of serotonin and dopamine. While this may be the case for both Tai-Chi and Yoga, the results of the meta-analysis were more convincing for the latter. The single study demonstrating significant positive effects of Tai-Chi on perceived stress, clearly denotes the need for further studies on the matter.

### 4.5. Study Strengths and Limitations

We used different parameters to help us/researchers better determine whether Tai Chi/Yoga can generate positive effects on the autonomic nervous system. The results of this current meta-analysis showed convergence in the results of the three parameters mutually provided strong support about the positive effects of TaiChi/Yoga on HRV. A limitation of the current study was that we only included manuscripts searched in English-based databases. There may have been a significant number of studies published in other languages that were left out of this meta-analysis. Another limitation was the combination of studies with Tai-Chi and Yoga in order to run the meta-analysis. Due to the limited number of published studies, it was not possible to analyze the separate effects of each modality on the HRV parameters. Thirdly, the technique of Yoga greatly varied in terms of cultures. Because most of selected studies did not note the specific naming of Yoga, we could not determine which type of Yoga is optimal for ANS and perceived stress. A final limitation was the heterogeneity of the samples used in the different studies included in the meta-analysis. We opted for not excluding studies based on participant characteristics because of the limited number of studies published on the subject. It is possible that the effects of Tai-Chi and Yoga may present differential benefits on HRV that could be moderated by individual characteristics (e.g., age, sex, gender, health history, etc.). In addition, the design of many of the studies included in the meta-analysis had significant flaws; including there were no participant blinding and no instructor blinding in all studies which might affect biases in expectation and in delivering the intervention, only 6/17 studies had allocation concealment, 9/17 of the studies lacked blinded assessors, there were high attribution rates in 9/17 studies, and only 9/17 studies used intention-to-treat analysis.

## 5. Conclusions

The results of this meta-analysis indicated a beneficial effect of Tai Chi/Yoga training on HRV parameters and perceived stress. The effects were small for nLF and nHF, moderate for LF/HF ratio, and large for perceived stress. Overall, the studies using Yoga were more efficacious in promoting changes in HRV parameters and perceived stress than those using Tai-Chi. Based on the results, it appears that at least 60 min/week of Yoga is necessary for reducing nLF and increasing nHF of the heart rate spectral power, as well as for reductions in perceived stress. Reductions in LF/HF ratio seem to occur with at least 90 min/week of Yoga. In relation to the benefits of Tai-Chi on HRV parameters and perceived stress, the results are inconclusive, given the minimal number of studies demonstrating significant results with several study and methodological limitations. Therefore, future studies with more rigorous design need to further examine whether Tai-Chi can consistently promote changes in HRV and perceived stress.

## Figures and Tables

**Table 1:** Features of randomized controlled trials.

Study	Participants	Duration (Weeks)	Intervention Protocol		Outcome Measured			Safety
			Experiment	Control	Time-Domain or Frequency Domain	Detection Method (Position)	Period Length	AE
Zheng et al. (2018) [38]	Healthy but stressed people, Mean age: 33.9 N = 55(27.5%), 11 M; 49 F	12 Weeks	5 × 60 min/week, Tai Chi	C1: 5 × 60 min/week, Other Gym exercise; C2: waitlist	LF, HF, LF/HF ratio, nLF, nHF; stress (PSS)	ECG (siting)	10 min	No
Lu et al. (2012) [39]	Middle-aged/elderly people, Mean age: 55 N = 50 (0%), 20 M; 30 F	12 weeks	7 × 40 min/week, Tai Chi	Unaltered lifestyle	LF, HF, LF/HF ratio, nLF, nHF	ECG (supine)	10 min	No
Audette et al. (2006) [40]	Elderly women, Mean age: 71.4 N = 34 (20.5%), 0 M; 34 F	12 weeks	3 × 60 min/week, Tai Chi	3 × 60 min/week, brisk walking	LF/HF ratio, nLF, nHF	ECG (NR)	5 min	No
Wong et al. (2018) [41]	Women with fibromyalgia, Mean age: 51 N = 37 (16.2%), 0 M; 37 F	12 weeks	3 × 55 min/week, Tai Chi	Unaltered lifestyle	nLF, nHF	The SA-200E model (supine), 12 breaths/min	5 min	No
Lin et al. (2015) [37]	Mental health professionals, Mean age: 30.92 N = 60 (0%), 12 M; 48 F	12 weeks	1 × 60 min/week, Yoga	Watching TV during a free tea time	LH, HF, LF/HF ratio, stress (the Chinese Version of Work-related Stress Scale)	HRV Monitor (NR)	NR	No
Santaella et al. (2011) [42]	Healthy elderly people, Mean age: 68 N = 30 (3.3%), 10 M; 19 F	16 weeks	2 × 30 min/week, Yoga	2 × 30 min/week, Stretching	LF, HF, nLF, nHF	ECG (sitting)	20 min	No
Cheema et al. (2013) [43]	university-based office employees Mean age: 38 N = 37 (8.1%), 7 M; 30 F	10 weeks	3 × 50 min/week, Hatha Yoga	Unaltered lifestyle	LnLF, LnHF, LnLH/LnHF ratio, Log RMSS, Log SDNN, PNN50	The Sphygmocor system (supine)	10 min	No
Huang et al. (2013) [44]	Female community residents Mean age: 45.8 N = 63 (1.6%), 0 M; 63 F	8 weeks	1 × 90 min/week, Yoga	Unaltered lifestyle	LF/HF ratio, nLF, nHF, Stress (PSS)	ECG (sitting)	5 min	No
Chu et al. (2015) [45]	Healthy women, Mean age: 26.21 N = 52 (11.5%), 0 M; 52 F	8 weeks	2 × 60min/week, Yoga	Unaltered level of physical activity	LF/HF ratio, nLF, nHF, SDNN, stress (PSS)	ECG (supine)	20 min	No
Jones et al. (2006) [46]	menopausal women Mean age:54.7 N = 355 (5.6%) 0M; 355 F	12 weeks	2 × 90 min/week + 20 min daily home practice, Yoga	C1: 3 × 40 min/week, other exercise C2: usual care	LH, HF, LF/HF ratio, nLF, nHF	ECG (sitting)	15 min	No
Wolever et al. (2012) [47]	highly stressed employees Mean age:42.9 N = 239(9.5%), 56 M; 183F	12 weeks	1 × 60 min/week, Yoga	C1: 14-h training in total, mindfulness C2: unaltered lifestyle	HRV Coherence ratio, RR interval stress (PSS)	emWave Ear Sensor (sitting)	10 min	No
Satyapriya et al. (2009) [48]	Pregnant women Mean age: 25.85 N = 122 (27.8%) 0 M; 122 F	16 weeks	3 × 120 min/week for Week 1 + 60min daily home practice for other 15 weeks (Yoga)	standard prenatal exercise	LF, HF, LF/HF ratio; stress (PSS)	ECG (NR)	5 min	No
Bowman et al. (1997) [49]	Healthy sedentary elderly subjects Mean age: 68 N = 40 (35%), 23 M; 17 F	6 weeks	2 × 45 min/week, Yoga	2 × 45 min/week, bicycle-base, aerobic training	HF	ECG (supine)	20 min	No
Zhou et al. (2018) [50]	Patients with NPC, Age range: 18–70 N = 114 (27.2%), 83 M; 31 F	19 weeks	5 × 60 min/week, Tai Chi during chemordiotherapy	Usual care during chemordiotherapy	nLF, nHF, nLF/nHF ratio	ECG (supine)	5 min	No
Chu et al. (2017) [51]	Sedentary women with depressive symptoms, Mean age: 32.7 N = 26 (23%), 0 M; 26 F	12 weeks	2 × 60 min/week, Yoga	Wait-list	nLF, nHF, LH/HF ratio, SDNN; stress (PPS)	ECG (supine)	20 min	No
Karishna et al. (2014) [52]	Patients with CHF Mean age:49.8 N = 130 (29.2%), 64 M; 28 F	12 weeks	3 × 60 min/week, Yoga during standard medical therapy	Standard medical therapy	LF/HF ratio, nLF, nHF	ECG (supine)	10 min	No
Telles et al. (2016) [53]	Patients with chronic low back Mean age: 35.6 N = 62 (46.8%), 32 M; 30 F	12 weeks	3 × 60 min/week for 2 weeks + daily home practice (10 weeks), Yoga	Standard care	LF/HF ratio, nLF, nHF, RMSSD	ECG (sitting)	5 min	No

Note: M = male; F = female; C1 = Control group 1; C2 = Control group 2; NPC = Nasopharyngeal Carcinoma; CHF = Chronic heart failure; ECG = Electrocardiogram;PSS-14 = Perceived Stress Scale; HRV = heart rate variability; LF = lower-frequency; HF = high-frequency; LF/HF ratio = low frequency to high frequency ratio; nLF = low-frequency normalized units; nHF = high-frequency normalized units; LnLF = natural logarithm of low frequency; LnHF = natural logarithm of high frequency; SDNN = Standard deviation of all NN intervals; RMSSD = Square root of the mean of the square of differences between adjacent NN intervals; PNN50 = percentage of absolute differences between successive normal RR intervals that exceed 50 ms; AE = adverse event.

**Table 2:** Study quality assessment.

Reference	Item 1	Item 2	Item 3	Item 4	Item 5	Item 6	Item 7	Item 8	Item 9	Sum Scores
Zheng et al. (2018) [38]	1	1	1	1	0	0	1	1	1	7
Lu et al. (2012) [39]	1	1	0	1	0	1	0	1	1	6
Audette et al. (2006) [40]	1	1	0	1	1	0	0	0	1	5
Wong et al. (2018) [41]	1	1	0	1	1	0	0	1	1	6
Lin et al. (2015) [37]	1	1	1	0	0	1	1	1	1	7
Santaella et al. (2011) [42]	1	1	0	1	0	1	0	1	1	6
Cheema et al. (2013) [43]	1	1	1	1	1	1	1	1	1	9
Huang et al. (2013) [44]	1	1	0	1	0	1	0	1	1	6
Chu et al. (2015) [45]	1	1	0	1	1	1	1	1	1	8
Jones et al. (2016) [46]	1	1	0	1	1	1	0	1	1	7
Wolever et al. (2012) [47]	1	1	0	1	0	1	1	1	1	7
Satyaprya (2009) [48]	1	1	1	1	1	0	0	1	1	7
Bowman et al. (1997) [49]	1	1	0	1	0	0	0	1	1	5
Zhou et al. (2018) [50]	1	1	1	1	0	0	1	1	1	7
Chu et al. (2017) [51]	1	1	0	1	1	0	1	1	1	7
Karishna et al. (2014) [52]	1	1	0	1	0	0	0	1	1	5
Telles et al. (2016) [53]	1	1	1	1	1	0	1	1	1	8

Note: Item 1 = eligibility criteria; Item 2 = random allocation; Item 3 = allocation concealment; item 4 = baseline equivalence; Item 5 = blinding of all assessors; Item 6 = retention rate of ≥85%; Item 7 = intention to treat analysis; Item 8 = between-group comparisons; Item 9 = point measures and measures of variability; “0” = unclear; “1” = clearly described item.
